# Quality of Available Cardiovascular Disease Knowledge Tools: A Systematic Review

**DOI:** 10.5334/gh.1446

**Published:** 2025-07-09

**Authors:** Michael A. Fajardo, Cassia Yung, Samuel Cornell, Rajesh Puranik, Anna L. Hawkes, Shiva Raj Mishra, Jenny Doust, Carissa Bonner

**Affiliations:** 1 Faculty of Medicine and Health, School of Public Health, University of Sydney, NSW, Australia; 2 Menzies Centre for Health Policy and Economics, University of Sydney, NSW, Australia; 3 Sydney Health Literacy Lab, University of Sydney, NSW, Australia; 4 School of Public Health, Faculty of Medicine, University of Queensland, Herston, QLD, Australia; 5 Australian Women and Girls’ Health Research (AWaGHR) Centre, School of Public Health, Faculty of Medicine, University of Queensland, Herston, QLD, Australia

**Keywords:** Systematic Review, Cardiovascular Disease, Health Literacy, Health Knowledge, Patient Health Questionnaire

## Abstract

**Background:**

Cardiovascular disease (CVD) is the leading cause of morbidity and mortality. Most people can reduce their CVD risk through lifestyle improvements and medication. Having low health literacy is a barrier to CVD prevention and management and is associated with worse health outcomes. Knowledge is a key component of health literacy, but there is no standard way for clinicians to assess this to tailor education about CVD. The aim of this review was to identify available CVD knowledge tests and evaluate their quality.

**Methods:**

Electronic database searches were conducted using Medline, CINAHL, PsycINFO and PsycTESTS between inception and October 2022. Identified tools were assessed using the Psychometric Grading Framework (PGF) to assess the quality of included tests.

**Results:**

A total of 28 studies were identified, of which 18 were original test development papers and 10 were language translation papers. The five most common domains were CVD risk factors, nutrition, heart physiology, physical activity, and treatment options. Three papers achieved an A grading on the PGF. Only one test provided a guide to classify patients based on the results.

**Conclusions:**

This review identified 15 additional knowledge assessment tools compared to previous research, including some available in multiple languages. Clinicians can access a wide range of CVD knowledge assessment tools to understand and respond to patient knowledge levels, but some are higher quality than others. Alternative tools may be needed to assess specific risk factor and condition knowledge. Further work is needed to tailor CVD knowledge tests for populations lower health literacy, and to validate the tests against health outcomes to improve clinical practice.

**PROSPERO:**

CRD42022370227

**What is Known:**

- Having low health literacy is associated with worse cardiovascular outcomes.
- Knowledge is a key component of health literacy, but there is no standard way to assess this for CVD.
- An assessment of the quality and reliability of CVD knowledge tests could support clinicians to tailor patient education to health literacy needs.

**What the Study Adds:**

- We identified 15 additional tests development papers that were not captured in earlier reviews of CVD knowledge tests.
- There are multiple high quality CVD knowledge tests available to clinicians, and tests available in different languages.
- These tests may be used to tailor patient education to individual health literacy needs.

## Introduction

Cardiovascular disease (CVD) is the leading cause of morbidity and mortality worldwide. Cardiovascular risk can be reduced through lifestyle changes and medication (1,2,3,4). Having low health literacy is a barrier to CVD prevention and management and is associated with worse health outcomes (5,6,7,8). This includes worse knowledge about risk factors and lower engagement with health professionals which contributes to greater hospital attendance, higher risk of stroke, lower quality of life, and greater risk mortality downstream (5,7,8).

Knowledge is a key component of health literacy in either context of understanding risk or self-management of an established disease. For example, disease-specific knowledge is essential for self-management as it enables independent decision making and behaviour change. Models of patient activation position knowledge as essential for increasing individual’s skills and confidence in managing their healthcare (9). Chronic care models also stress the importance of building patient’s understanding of their health condition, allowing them to take a leading role in their self-management (10). What these models have in common is that patients require disease-specific knowledge to better understand and manage their condition.

Despite the importance of disease-specific knowledge in all aspects of CVD prevention and management, there is no standard way for clinicians to assess this to tailor patient education about CVD. Previous reviews of CVD knowledge tests focused on single conditions (e.g. heart failure) and specific languages (e.g. Spanish) using narrowly defined search terms. Further, the quality of these tools was not assessed in previous reviews, so it is unclear to users which tools have been developed using rigorous processes to ensure reliable and valid results.

The aim of this paper was to identify and evaluate currently available CVD knowledge assessments to identify reliable and valid tools. This paper will extend on previous research (11,12) by exploring the availability and psychometric properties of CVD related knowledge instruments, using a more comprehensive search and evaluation method.

## Methods

This review process was based on the Cochrane Handbook for Systematic Reviews (13) and is registered in PROSPERO (CRD42022370227).

### Inclusion and exclusion criteria

Criteria were based on the PICO format, however given the nature of test development, comparators were not considered. We were interested in patients with CVD or individuals in the general population that were not trained in a health profession (Population). We were interested in CVD knowledge test development (Intervention) and their psychometric properties (Outcome). We included primary studies that developed and assessed knowledge of CVD in clinical or general populations. Knowledge tests for health professionals were not considered. CVD knowledge was defined broadly to include basic CVD physiology, prevention, and management (clinical and behavioural risk factors). Eligibility criteria included: the tool is free for public use, the paper provides information on reliability and validity, and the paper is written in English (but the knowledge test did not have to be written in English).

### Search strategy

Three core search concepts and their related search terms were reviewed by a cardiologist and CVD patient education expert. Each concept was combined with a proximity operator (e.g. W3 or N3) as assessment tools typically have descriptive names. The three search concepts were i) Heart, ii) Knowledge and iii) Tests. Heart consisted of the terms: “heart”, “cardiovascular”, and “vascular”. Knowledge consisted of the terms: “knowledge”, “comprehension”, “understanding”, “awareness”, “health literacy”, “literacy” and “self-management”. Test consists of the terms: “test”, “exam*”, “asses*”, “evaluat*”, “check” and “tool”. Scopus, Medline, CINAHL, PsycINFO and PsycTESTS were searched between inception and October 2022 (see Appendix 1 for full search strategies). Search results were uploaded to Covidence where screening and full text review were completed. Two similar previous reviews (11,12) were also identified and whose reference lists also were added to the final list.

### Search methods for identification of studies

Abstract screening was performed by two authors (MF, SC), and discrepancies in screening were addressed at full text review. Two authors (MF, SC) completed full text assessment and a third author (CB) resolved discrepancies. Initial full text agreement prior resolving discrepancies was 86% (Cohen’s κ =.74).

### Risk of bias

A data extraction form was created for the purposes of this review (see Appendix 2). The psychometric grading framework (14) (PGF) was used to assess the quality of these papers. The PGF is based on commonly used statistical test and values recommended by psychologists and biostatisticians. It provides an evaluation grade between A to D based on the tool’s content, construct and criterion validities, test-retest reliability, and internal consistency. For each of these sections, the PGF provides specific criteria per grade (e.g., for internal consistency, an A grade requires Cronbach’s alpha to be greater than or equal to .90). Inter-rater reliability was not considered as it was not applicable for knowledge scales. Additionally, knowledge scale development may involve testing group differences to establish criterion validity, such as those diagnosed with CVD compared to those who have not been (the assumption being that those with CVD would know more about their condition). The PGF does not differentiate between confirmatory and exploratory factor analysis for the purpose of assessing construct validity (14). When determining the overall grade, papers with insufficient information to evaluate a specific PGF domain were considered a “D” grade whereas those with relevant information which did not fit PGF criteria were considered case-by-case (e.g., when translation studies cited studies that demonstrated criterion validity previously). An overall A grade means the psychometric strength of the instrument is ‘good’ and can be given to an instrument with mostly A’s and some B’s and or C’s. The test development is also described and discussed in the context of educational test development. This included pilot testing, item writer training, predetermined pass levels, qualitative answer rubrics, expert input and type of questions (15).

### Data extraction and synthesis

Data extraction was performed by one author (MF) and then reviewed by a second author (CY). Corresponding authors were contacted for missing data. Of the five emails contacted, one was no longer active and four have not returned responses by the submission of this manuscript. Pooled estimates were not considered appropriate for this review. Descriptive statistics were used to summarise characteristics. Knowledge test domain areas were extracted as defined by study authors, thematically organised and then applied to all included knowledge tests to obtain a frequency table of areas assessed. All data and supporting materials have been provided with the published article.

## Results

### Search results

**Figure 1:**
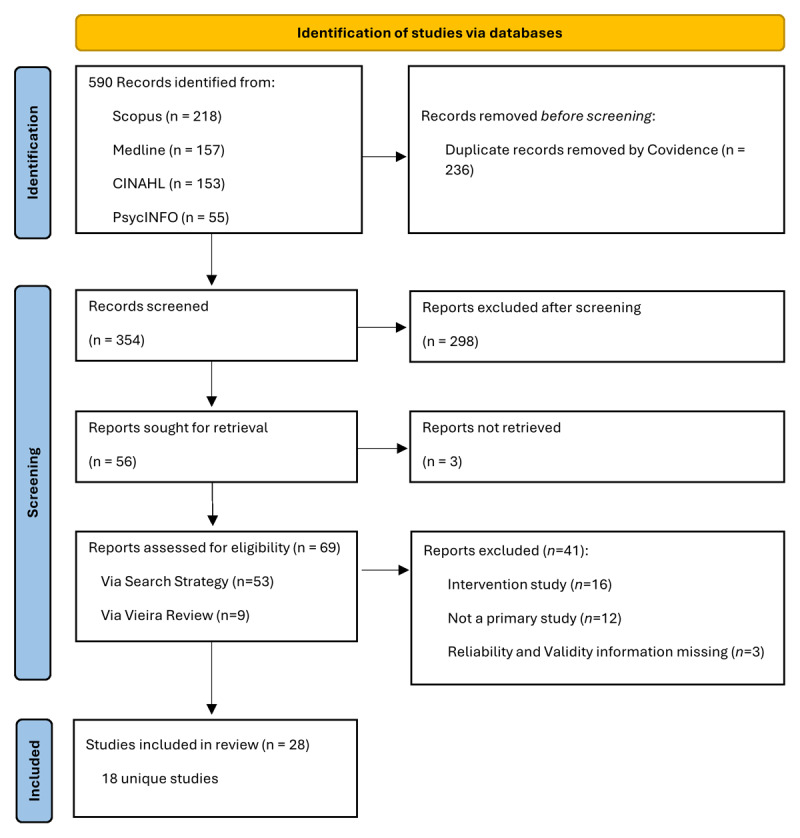
PRISMA Flow Diagram.

Figure 1 shows that this search yielded a total of 28 papers: 18 development and 10 translation studies (i.e. a study that validated a knowledge test that was originally written in English then translated to another language or vice versa). The final list can be found in Table 3. We identified 15 additional papers that were not captured in earlier reviews (11,12).

### Knowledge test characteristics

Table 1 describes the key characteristics of the CVD knowledge tests and samples used to assess the psychometric properties of the scale. All studies focused on adults except Cecchetto and Pellanda (16) which was the only child knowledge scale. Table 2 describes the domains of each knowledge test by study. Knowledge of risk factors for CVD was the most common content area measured (10 development studies, nine translated language studies), followed by nutrition and diet (10 development studies, seven translated language studies), medical knowledge/pathophysiology of the heart (nine development studies, six translated language studies) physical activity and exercise (six development studies, five translated language studies), treatment options (eight development studies, one translated language study), symptoms and signs of heart failure/disease (five development studies, two translated language studies), self-care and management (seven development studies, zero translated language studies), psychosocial risk (one development study, four translated language studies) and epidemiology knowledge of CVD (one development study, one translated language study). All tests were multiple choice items (either true/false or select best answer). All scoring for tests was predetermined and typically followed one point per correct answer where higher scores indicated higher knowledge levels.

**Table 1:** Scale and sample characteristics of included studies.


	DEVELOPMENT	TRANSLATED LANGUAGE	TOTAL

**Question Length**			

Mean	19	21.5	20

Median	21.6	21.5	21.5

Minimum	5	12	5

Maximum	64	30	64

**Question Types (Count)**			

True/False (Yes/No) + IDK	7	6	13

Multiple Choice	9	3	12

Combination	1	0	1

Likert Scale	1	1	2

**Sample Type (Count)**			

Clinical	13	9	22

General Population	4	1	5

Pre-clinical	1	0	1

**Gender Male Mean %**	0.63^a^	0.55	0.56

**Gender Male Median %**	0.57^a^	0.51	0.55

**Mean Age per Sample**			

Mean	57.8^b^	61.0^a^	59.1^c^

Median	60.5^b^	60.7^a^	60.6^c^


^a^Missing data = 1 study; ^b^Missing data = 5 studies; ^c^Missing data = 6 studies.

**Table 2:** Domain areas covered by each CVD knowledge scale, as defined by authors sorted by most frequent domain areas.


STUDY		RISK FACTORS	NUTRITION/DIET	MEDICAL KNOWLEDGE/PATHOPHYSIOLOGY OF <3	PHYSICAL ACTIVITY/EXERCISE	TREATMENT OPTIONS	SYMPTOMS	SELF-CARE & MANAGEMENT	PSYCHO SOCIAL RISKS	EPIDEMIO LOGICAL KNOWLEDGE	STRESS	HEART FAILURE SPECIFIC HEALTH LITERACY	CHD, CONTRACEPTIVES AND PREGNANCY	DIABETES AND CVD	RHEUMATOID ARTHRITIS SPECIFIC RISK FACTORS	PRE-HOSPITAL ADMISSION	THOUGHTS & ACTIONS TOWARDS HEART FAILURE	HYPERTENSION	FEMALE SPECIFIC CHD KNOWLEDGE

Development Studies	Bergman 2011 (27)	✓	✓	✓			✓			✓									

	Bonin 2014 (28)	✓		✓		✓	✓	✓											

	Butts 2018 (17)		✓			✓	✓	✓											

	Cecchetto 2014 (16)	✓	✓		✓			✓											

	Ghisi 2010b (29)	✓	✓	✓	✓	✓													

	Gwadry-Sridhar 2003 (30)	✓	✓		✓	✓													

	John 2009 (19)	✓													✓				

	Kato 2013 (31)			✓		✓	✓	✓											

	Khazaei 2018 (23)																✓		

	Lainscak 2005 (32)		✓	✓				✓											

	Reilly 2009 (33)		✓	✓	✓	✓		✓											

	Santos 2018 (34)	✓		✓	✓	✓		✓											

	Schapira 2012 (35)																	✓	

	Smith 1991 (36)	✓	✓	✓	✓				✓		✓								

	Thanavaro 2010 (21)																		✓

	van der Wal 2005 (37)		✓	✓		✓	✓												

	Wagner 2005 (38)	✓																	

	Woringer 2017 (39)	✓	✓																

Language Translation Studies	Frayssac 2017 (18)	✓													✓				

	Ghisi 2010a (40)	✓	✓	✓												✓			

	Ghisi 2018 (41)	✓	✓	✓	✓				✓										

	Lima 2022 (42)	✓	✓	✓			✓			✓									

	Omovvat 2022 (43)	✓	✓		✓	✓			✓										

	Saffi 2013(44)	✓	✓	✓	✓		✓				✓								

	Santos 2019 (45)	✓	✓	✓	✓				✓										

	Yang 2020 (22)	✓	✓	✓	✓				✓										

	Yue 2016 (46)											✓							

	Zehirlioglu 2020 (47)	✓												✓					


### Test development characteristics

All studies except Butts et al (17) described an acceptable level of expert input as per the PGF towards the development of scale items, however this was because Butts et al (17) was a revalidation study of the Atlanta Heart Failure Knowledge Test Version 2, so expert input was already accounted for. For language translated studies, the translations were completed by bilingual researchers. No study clearly indicated any authors received item writing training (15). All studies except Butts et al (17) and Frayssac et al (18) described pilots. Pilots varied in terms of samples used (general population, clinical, professionals), implementation (single samples, or follow-up samples) and reported outcomes for pilots (clarity, content, understanding). No study identified an a priori level of desired or acceptable knowledge. One study, John et al (19) had parallel forms (two versions of the same form that can be used to minimise practice effects when testing over two time periods). No included study correlated knowledge test score with patient outcomes.

### Psychometric properties

Table 3 provides the gradings from the PGF per study ordered by surname within unique and translated language study groups. Two unique studies, Khazaei et al (20) and Thanavaro et al (21), one translated language study, Yang et al (22) obtained an overall A rating. Of the remaining studies, four received an overall B rating, 12 received an overall C rating, and nine received an overall D rating.

**Table 3:** Quality ratings for each CVD knowledge scale.


STUDY		CONSTRUCT VALIDITY	CRITERION VALIDITY	INTERNAL CONSISTENCY	TEST-RETEST RELIABILITY	OVERALL RATING

Unique	Bergman 2011 (27)	A	†	C	†	C

	Bonin 2014 (28)	D	†	C	A	C

	Butts 2018 (17)	A	C	B	†	B

	Cecchetto 2014 (16)	D	†	A	A	B

	Ghisi 2010b (29)	D	†	D	A	C

	Gwadry-Sridhar 2003 (30)	†	†	C	†	D

	John 2009 (19)	D	†	D	D	D

	Kato 2013 (31)	D	†	C	†	D

	Khazaei 2018 (20)	A	†	B	A	A

	Lainscak 2005 (32)	†	†	C	†	D

	Reilly 2009 (33)	A	†	C	†	C

	Santos 2018 (34)	D	†	C	A	C

	Schapira 2012 (35)	C	†	*	†	D

	Smith 1991 (36)	†	*	B	†	C

	Thanavaro 2010 (21)	A	A	C	B	A

	van der Wal 2005 (37)	*	*	D	†	D

	Wagner 2005 (38)	†	C	C	A	C

	Woringer 2017 (39)	D	†	C	†	D

Translation	Frayssac 2017 (18)	D	†	C	B	C

	Ghisi 2010a (40)	D	†	D	B	C

	Ghisi 2018 (41)	D	†	C	B	C

	Lima 2022 (42)	A	†	D	A	B

	Omovvat 2022 (43)	*	†	C	B	C

	Saffi 2013 (44)	D	†	C	†	D

	Santos 2019 (45)	†	*	D	A	C

	Yang 2020 (22)	A	*	A	B	A

	Yue 2016 (46)	D	†	B	A	B

	Zehirlioglu 2020 (47)	*	†	†	†	D


* Information available but does not fit PGF criteria; † insufficient information to evaluate.

## Discussion

### Key findings

This review identified a total of 28 papers, 18 of which were development papers and 10 were language translation papers. This review added 15 new papers when compared to previous reviews conducted by Vieira et al (11) and da Rocha et al (12). Collectively, knowledge tests assessed a wide variety of domains within the context of CVD physiology, assessment, and management. The five most frequent domains assessed by included tools were Risk Factors, Nutrition/Diet, Medical Knowledge of Heart/Pathophysiology Physical Activity/Exercise and Treatment Options. Uncommon domains included thoughts and feelings around heart failure and female specific CVD risk factors that may highlight gaps in the literature around heart knowledge.

The psychometric quality of these tools is quite variable, and none were validated against health outcomes. Khazeai et al (23), Thanavaro et al (21) and Yang et al (22) were assessed to have an A rating. As our findings are limited to papers related to the search criteria used, it resulted in a more comprehensive list of knowledge tests than previous reviews. Other tests, however, did not appear in our search, namely tests that are specific to nutrition (24,25). Readers seeking CVD knowledge tests for specific patient populations or specific risk factors may need to conduct additional searches using relevant terms to find additional tools.

### Implications and future directions

CVD knowledge test developers are recommended to have questionnaire item writing training (15), and trained item writers should be involved early in the test development phase (26). Pre-determining an acceptable level of CVD knowledge may support clinical use by providing a minimum level of knowledge that is acceptable for a specific purpose, such as informed consent. This pre-determined level, however, would need to be balanced with the need for variability in measures for statistical development purposes, which may be the reason why very few papers discussed this. Assessing knowledge scales against health outcomes is typically done separately to the initial test development once the traditional psychometric properties of the assessment tool are sound. If validation against health outcomes was conducted as part of the psychometric properties, it would provide a more direct clinical utility to understanding the construct of knowledge and its impact on health outcomes earlier in the process. For example, measuring current self-help behaviours and correlating this variable with self-help domains in knowledge scales.

Given the impact of disease specific knowledge on self-management, clinical uses for CVD knowledge tests can help tailor to patient needs (10). For example, a CVD knowledge test after a cardiac event may be helpful to identify additional education support needs for self-management; whereas a CVD knowledge test in an asymptomatic individual may be an awareness raising tool to prompt a CVD risk assessment with their doctor. Only seven identified papers included self-management questions, which may limit the pool of tests that are useful in cardiovascular settings. Clinicians may benefit from a centralised bank of CVD knowledge questions, with domain specific areas that can be mixed and matched for different inpatient and outpatient settings. Validated questions may be used more qualitatively in practice, to enable clinicians to use these tools in a flexible manner tailored to individual patient needs. Translation services could allow these tools to be adapted to additional languages around the world. Future research could investigate how validated questionnaire items need to be tailored to patient populations with different health conditions, socio-economic position and health literacy status. The understandability and face validity of the questions for patients with lower health literacy should also be considered.

### Strengths and limitations

Drawing on educational test methods provides an additional lens to evaluate the quality of disease-specific knowledge tests (15). However, the PGF may not be adequately equipped to address the difference between a confirmatory factor analysis and exploratory factor analysis, which negatively impacted some ratings. Final PGF ratings permit an overall A rating even when instruments receive B or C grades in specific domains (but not D grades), acknowledging that variability or omission in some domains may not necessarily render an instrument inadequate. The search strategy was more comprehensive than earlier reviews but did not specifically search for risk factor knowledge tests or specific cardiovascular populations. Furthermore, publication bias may be considered given this review did not source grey literature, however, given the variability of PGF ratings, the impact of publication bias could be considered minimal.

## Conclusions

This paper shows there are many readily available and psychometrically sound CVD knowledge tests that researchers and clinicians can access, and we have identified higher quality tools using a comprehensive educational framework. The identified tests may be used to facilitate tailored patient education to better support the prevention and management of CVD. Future work is needed to tailor CVD knowledge tests for populations lower health literacy, and to validate the tests against health outcomes to improve clinical practice.

## Additional Files

The additional files for this article can be found as follows:

10.5334/gh.1446.s1 Appendix 1. Table S1 Search Strategy.

10.5334/gh.1446.s2 Appendix 2. Table S2 Data Extraction Form.
