# Supplementary material for: Quality of Available Cardiovascular Disease Knowledge Tools: A Systematic Review
Source: Glob Heart. 2025 Jul 9;20(1):59. doi: 10.5334/gh.1446 (PMC12247824; doi:10.5334/gh.1446)
Supplement: Appendix 1. — Table S1 Search Strategy. [file gh-20-1-1446-s1.pdf]

Appendix 1

Table S1. Search Strategy

Database	Search Strategy	Limiters
Scopus	TITLE-ABS-KEY (heart OR cardiovascular OR vascular) W/3 (knowledge OR comprehension OR understanding OR awareness OR “health literacy” OR literacy OR self?management) W/3 (test OR exam* OR asses* OR evaluat* OR check OR tool) AND (LIMIT-TO (DOCTYPE , "ar")) AND (LIMIT-TO (LANGUAGE , "English"))	English, Article
Medline	((heart OR cardiovascular OR vascular) adj3 (knowledge OR comprehension OR understanding OR awareness OR health literacy OR literacy OR self?manage*) adj3 (test OR exam* OR asses* OR evaluat* OR check OR tool))	English; Humans
CINAHL	(heart N3 knowledge N3 exam*) OR<br>(heart N3 knowledge N3 asses*) OR<br>(heart N3 knowledge N3 evaluat*) OR<br>(heart N3 knowledge N3 check) OR<br>(heart N3 knowledge N3 tool) OR<br>(heart N3 comprehension N3 test) OR<br>(heart N3 comprehension N3 exam*) OR<br>(heart N3 comprehension N3 asses*) OR<br>(heart N3 comprehension N3 evaluat*) OR<br>(heart N3 comprehension N3 check) OR<br>(heart N3 comprehension N3 tool) OR<br>(heart N3 understanding N3 test) OR<br>(heart N3 understanding N3 exam*) OR<br>(heart N3 understanding N3 asses*) OR<br>(heart N3 understanding N3 evaluat*) OR<br>(heart N3 understanding N3 check) OR<br>(heart N3 understanding N3 tool) OR<br>(heart N3 awareness N3 test) OR<br>(heart N3 awareness N3 exam*) OR<br>(heart N3 awareness N3 asses*) OR<br>(heart N3 awareness N3 evaluat*) OR<br>(heart N3 awareness N3 check) OR<br>(heart N3 awareness N3 tool) OR<br>(heart N3 "health literacy" N3 test) OR<br>(heart N3 "health literacy" N3 exam*) OR<br>(heart N3 "health literacy" N3 asses*) OR<br>(heart N3 "health literacy" N3 evaluat*) OR<br>(heart N3 "health literacy" N3 check) OR<br>(heart N3 "health literacy" N3 tool) OR<br>(heart N3 literacy N3 test) OR<br>(heart N3 literacy N3 exam*) OR<br>(heart N3 literacy N3 asses*) OR	English language, Academic Journals

(heart N3 literacy N3 evaluat*) OR
(heart N3 literacy N3 check) OR
(heart N3 literacy N3 tool) OR
(heart N3 self?manage* N3 test) OR
(heart N3 self?manage* N3 exam*) OR
(heart N3 self?manage* N3 asses*) OR
(heart N3 self?manage* N3 evaluat*) OR
(heart N3 self?manage* N3 check) OR
(heart N3 self?manage* N3 tool) OR
(cardiovascular N3 knowledge N3 test) OR
(cardiovascular N3 knowledge N3 exam*) OR
(cardiovascular N3 knowledge N3 asses*) OR
(cardiovascular N3 knowledge N3 evaluat*) OR
(cardiovascular N3 knowledge N3 check) OR
(cardiovascular N3 knowledge N3 tool) OR
(cardiovascular N3 comprehension N3 test) OR
(cardiovascular N3 comprehension N3 exam*) OR
(cardiovascular N3 comprehension N3 asses*) OR
(cardiovascular N3 comprehension N3 evaluat*) OR
(cardiovascular N3 comprehension N3 check) OR
(cardiovascular N3 comprehension N3 tool) OR
(cardiovascular N3 understanding N3 test) OR
(cardiovascular N3 understanding N3 exam*) OR
(cardiovascular N3 understanding N3 asses*) OR
(cardiovascular N3 understanding N3 evaluat*) OR
(cardiovascular N3 understanding N3 check) OR
(cardiovascular N3 understanding N3 tool) OR
(cardiovascular N3 awareness N3 test) OR
(cardiovascular N3 awareness N3 exam*) OR
(cardiovascular N3 awareness N3 asses*) OR
(cardiovascular N3 awareness N3 evaluat*) OR
(cardiovascular N3 awareness N3 check) OR
(cardiovascular N3 awareness N3 tool) OR
(cardiovascular N3 "health literacy" N3 test) OR
(cardiovascular N3 "health literacy" N3 exam*) OR
(cardiovascular N3 "health literacy" N3 asses*) OR
(cardiovascular N3 "health literacy" N3 evaluat*) OR
(cardiovascular N3 "health literacy" N3 check) OR
(cardiovascular N3 "health literacy" N3 tool) OR
(cardiovascular N3 literacy N3 test) OR
(cardiovascular N3 literacy N3 exam*) OR
(cardiovascular N3 literacy N3 asses*) OR
(cardiovascular N3 literacy N3 evaluat*) OR
(cardiovascular N3 literacy N3 check) OR
(cardiovascular N3 literacy N3 tool) OR
(cardiovascular N3 self?manage* N3 test) OR
(cardiovascular N3 self?manage* N3 exam*) OR

(cardiovascular N3 self?manage* N3 asses*) OR
(cardiovascular N3 self?manage* N3 evaluat*) OR
(cardiovascular N3 self?manage* N3 check) OR
(cardiovascular N3 self?manage* N3 tool) OR
(vascular N3 knowledge N3 test) OR
(vascular N3 knowledge N3 exam*) OR
(vascular N3 knowledge N3 asses*) OR
(vascular N3 knowledge N3 evaluat*) OR
(vascular N3 knowledge N3 check) OR
(vascular N3 knowledge N3 tool) OR
(vascular N3 comprehension N3 test) OR
(vascular N3 comprehension N3 exam*) OR
(vascular N3 comprehension N3 asses*) OR
(vascular N3 comprehension N3 evaluat*) OR
(vascular N3 comprehension N3 check) OR
(vascular N3 comprehension N3 tool) OR
(vascular N3 understanding N3 test) OR
(vascular N3 understanding N3 exam*) OR
(vascular N3 understanding N3 asses*) OR
(vascular N3 understanding N3 evaluat*) OR
(vascular N3 understanding N3 check) OR
(vascular N3 understanding N3 tool) OR
(vascular N3 awareness N3 test) OR
(vascular N3 awareness N3 exam*) OR
(vascular N3 awareness N3 asses*) OR
(vascular N3 awareness N3 evaluat*) OR
(vascular N3 awareness N3 check) OR
(vascular N3 awareness N3 tool) OR
(vascular N3 "health literacy" N3 test) OR
(vascular N3 "health literacy" N3 exam*) OR
(vascular N3 "health literacy" N3 asses*) OR
(vascular N3 "health literacy" N3 evaluat*) OR
(vascular N3 "health literacy" N3 check) OR
(vascular N3 "health literacy" N3 tool) OR
(vascular N3 literacy N3 test) OR
(vascular N3 literacy N3 exam*) OR
(vascular N3 literacy N3 asses*) OR
(vascular N3 literacy N3 evaluat*) OR
(vascular N3 literacy N3 check) OR
(vascular N3 literacy N3 tool) OR
(vascular N3 self?manage* N3 test) OR
(vascular N3 self?manage* N3 exam*) OR
(vascular N3 self?manage* N3 asses*) OR
(vascular N3 self?manage* N3 evaluat*) OR
(vascular N3 self?manage* N3 check) OR
(vascular N3 self?manage* N3 tool)

PsycINFO	((heart OR cardiovascular OR vascular) adj3 (knowledge OR comprehension OR understanding OR awareness OR health literacy OR literacy OR self?manage*) adj3 (test OR exam* OR asses* OR evaluat* OR check OR tool))	English, Human, Peer Reviewed Journals
PsycTEST	((heart OR cardiovascular OR vascular) adj3 (knowledge OR comprehension OR understanding OR awareness OR health literacy OR literacy OR self?manage*) adj3 (test OR exam* OR asses* OR evaluat* OR check OR tool))	English, Human
