# Supplementary material for: Quality of Available Cardiovascular Disease Knowledge Tools: A Systematic Review
Source: Glob Heart. 2025 Jul 9;20(1):59. doi: 10.5334/gh.1446 (PMC12247824; doi:10.5334/gh.1446)
Supplement: Appendix 2. — Table S2 Data Extraction Form. [file gh-20-1-1446-s2.pdf]

Appendix 2

Table S2. Data Extraction Form

	Data	Description	How data is recorded
Descriptive Information	Name of Instrument	Title of the heart knowledge test	Text entry (string)
	Norming Population: Clinical or General		Multiple Choice:<br>☐ General<br>☐ Clinical
	Norming Population: Descriptive	Include description of Country of Origin, ethnicity (e.g. representativeness of country of origin via Census data), age mean/median and range, Education levels, Occupation; Type of Clinical population	Text entry (string)
	Test Assessment Focus	What is the test actually assessing, e.g. CHD knowledge, Self-Care knowledge, Heart Disease Knowledge	Text entry (string)
	Test Administration	How is the test administered?	Multichoice
	Response style	How were respondents expected to respond to test items	Multichoice (Check all that apply):<br>☐ True/False<br>☐ Multiple Single Choice<br>☐ Multichoice<br>☐ Open Ended<br>☐ Rank Order
Test Development / RoB Type	Item Length per Response Style	Corresponds to Response Style	Text entry (numeric)
	Total Item Length	Total of all items per instrument irrespective of response style	Text entry (numeric)
	Expert Input on Test Items	Did the test items have expert input	Multiple Choice:<br>☐ Yes<br>☐ No
	Type of Expert Input	If there was expert input, what type of expert?	Multichoice (Check all that apply):<br>☐ Cardiologist<br>☐ Nurse Practitioner<br>☐ Other: ___
	Item Writer Training	Were test item writers trained?	Multiple Choice:<br>☐ Yes<br>☐ No/Unclear
	Pilot	Were test items piloted	Multiple Choice:

			☐ Yes<br>☐ No/Unclear
	Pilot Feedback	What feedback was provided by respondents to test developers after a pilot	Select: None OR Text entry (string)
	Predetermined "pass" level	Did developers consider a minimum level of understand or similar	Multiple Choice:<br>☐ Yes<br>☐ No/Unclear
	Qualitative Answer Standardisation	Did developers provide a standardised approach to grade qualitative responses?	Multiple Choice:<br>☐ Yes<br>No/Unclear
	Other TD/RoB Info	Other information relevant to understanding the test development/RoB to inform critical appraisal of this process	Text entry (string)
Reliability statistics	Test-retest reliability	What was the test-retest correlation	Text entry (numeric)
	Test-retest reliability time frame	How long was it between the test and retest	Test entry (string)
	Cronbach's Alpha (Internal Consistency)	What was the Cronbach's alpha for the assessment	Text entry (numeric)
	Validity Assessment	Describe any attempts to validate scores; I doubt this will be common though given "knowledge" assessments are inherently valid if they are reliable.	Text entry (string)
	Item Response Analysis	Did researchers employ Item Response Theory to individual analyse the data	Multiple Choice:<br>☐ Yes<br>☐ No
	Other information	Other important aspects of the test development that would contribute to its interpretation (e.g. if there are sub scales with their own Cronbach's alpha or different types of test-retest reliabilities)	Text entry (string)
